# Esophagectomy in Octogenarians in the Minimally Invasive Era: A NSQIP Analysis of Surgical Outcomes

**DOI:** 10.3390/jcm15197590

**Published:** 2026-09-30

**Authors:** Joshua Xu, Mary Hou, Estifanos Debru, Neal Church, Philip Mitchell, Daniel Meyer

**Affiliations:** Department of Surgery, University of Calgary, North Tower 1014, 1441 29th Street NW, Calgary, AB T2N 4J8, Canada

**Keywords:** esophagectomy, octogenarians, MIE

## Abstract

**Background:** Esophagectomy carries substantial morbidity and mortality with advanced age being a known risk factor. This study aims to evaluate the outcome of esophagectomy in octogenarians in the context of increasing adoption of minimally invasive esophagectomy (MIE). **Methods:** A retrospective cohort study was conducted using the National Surgical Quality Improvement Program database. Patients undergoing esophagectomy were stratified by age (<80 and ≥80). Univariate and multivariable analysis were used to assess the age and patient-dependent factors with surgical outcomes. **Results:** Among 11,210 patients identified who underwent esophagectomy, 447 were octogenarians. Overall, octogenarians experienced higher rates of 30-day mortality (*p* = 0.004), LOS (*p* = 0.0001), and serious complications (*p* = 0.001). Within the subset of MIE patients, morbidity (*p* = 0.001) and mortality (*p* = 0.051) remain elevated but significance was not reached for LOS (*p* = 0.116) between the two age groups. Multivariable analysis confirmed that age ≥80 was independently associated with serious complications (*p* = 0.008), readmission (*p* = 0.015), and extended LOS (*p* = 0.001). However, it was not an independent predictor of reoperation or anastomotic leak. MIE significantly decreased the rate of overall complications and extended LOS with a trend towards decreased mortality (*p* = 0.065). **Conclusions:** Octogenarians experience significantly higher morbidity, mortality, and post-surgical care needs. While age is a risk factor, patient-specific comorbidities and frailty are critical determinants of outcome. Furthermore, surgical approaches have a significant impact on the outcome of patients post-esophagectomy with MIE narrowing the outcome gap between the two age groups. These findings underscore the necessity for meticulous patient selection, preoperative optimization, and shared decision-making when considering esophagectomy in this growing demographic.

## 1. Introduction

Esophageal cancer is the eighth most common cancer worldwide and sixth in cancer-related mortality [1] with an estimated 511,000 new cases and 445,000 deaths globally in 2022 [2]. In the United States, the incidence is projected to exceed 22,000 cases in 2025. With the median age of diagnosis at 69 and 29.3% of new cases affecting patients above the age of 75, esophageal cancer disproportionally affects older adults [3].

Treatment of esophageal cancer is multidisciplinary, involving surgery, medical and radiotherapy, frequently involving all three modalities [4,5,6]. However, esophagectomy itself carries substantial perioperative risk, with overall complication rates of 42–65% and 90-day mortality reported at 4.5% in contemporary series [7].

Advanced age is a reported risk factor for post-operative morbidity and mortality following esophagectomy [8]. With the rapid shift toward an aging population and the increasing surgical needs by elderly patients [7], understanding outcomes of esophagectomy in octogenarians has become critically important for shared decision-making and informed patient choice [9]. Compared to patients under 80, octogenarians are at higher risk of post-surgical morbidity and mortality that increases progressively with age [8]. 30-day mortality has been described at 0.6% for patients <70 years old, 3.2% in patients 70–79 and 6.3% in octogenarians who also experience increased rates of complications at an odds ratio of 1.67 [10,11].

Currently, there is conflicting evidence in published literature regarding age as an independent variable in the outcome of esophagectomy [4,12,13]. These studies are limited by sample sizes, single center experiences, and heterogenous patient selection. Indeed, there have been past attempts to elucidate the outcome of elderly patients through large-scale national NSQIP dataset from 2005 to 2014, showing age independently associated with adverse outcomes including mortality [14]. However since then, there has been rapid adoption of minimally invasive approaches in esophagectomy that significantly improved mortality and morbidity in patients, with increase in minimally invasive or robotic-assisted esophagectomies of 49% and 704%, respectively, between 2010 and 2019 [15,16]. There is also evidence to suggest that the minimally invasive procedures may attenuate age-related differences in operative outcomes, including one study demonstrating no significant difference in overall survival after propensity score matching in patients above or below 75 years of age [17]. The widespread adoption of minimally invasive approaches in recent years necessitates an update to existing literature [7].

The current study aims to fill this gap by firstly evaluating practice patterns and approach to esophagectomy in patients over 80 years old. Secondly, the study assesses postoperative complications, including 30-day morbidity, reintervention, reoperation, and mortality rates. By elucidating the risks and realistic expectations of surgical intervention in this age group, our findings may guide clinicians in patient selection, preoperative optimization, and shared decision-making for octogenarians with esophageal cancer.

## 2. Materials & Methods

This retrospective cohort study utilized data from the National Surgical Quality Improvement Program (NSQIP) Participant Use File (PUF), a premier clinical surgical dataset in North America that aggregates over one million annual cases from accredited centers. These institutions adhere to rigorous international standards, with data collected prospectively by professional reviewers under HIPAA-compliant protocols. Data integrity is maintained through frequent audits, targeting a disagreement rate of no more than 5%. Given that the NSQIP database consists of de-identified, anonymous information, formal ethics approval was not required for this analysis.

All patients in the NSQIP database aged 18 or over undergoing esophagectomy for all indications were considered in this retrospective cohort study. Patients under age 18 were excluded from this analysis. Included patients were divided into two cohorts: <80 cohort if age was less than 80 at the time of surgery or octogenarians if age was ≥80.

Data collected included patient, operative, and post-operative factors. Patient factors included age, sex, race, height, weight, BMI, smoking status, functional status, American Society of Anesthesiologists (ASA) physical status classification, and comorbidities including diabetes, chronic obstructive pulmonary disease (COPD), hypertension, history of falls, dementia, renal dysfunction including pre-operative kidney injury or dialysis requirement, chronic immunosuppressive therapy, bleeding disorders, heart failure, ascites, or disseminated cancer. Technical factors included the year of the procedure, acuity of the procedure, unplanned conversions from minimally invasive to open, operative length, and primary procedure type including operative approach was obtained with the following standardized esophagectomy CPT codes: 43107, 43108, 43286, 43117, 43118, 43112, 43113, and 43288. Patients undergoing esophagectomy that did not have an immediate reconstruction were excluded. Use of a robot was also extracted and cross-referenced to CPT codes. The extended esophagectomy PUFs from the included years were also accessed to determine the presence of anastomotic leakage and management utilized, exposure to neoadjuvant radiation and/or chemotherapy within 90 days of the procedure.

Specific postoperative complications that were assessed included superficial and deep surgical site infection, organ space infection, wound disruption, urinary tract infection, pneumonia, Clostridium difficile infection, septic shock, bleed requiring transfusion, reintubation, failure to wean ventilator, postop acute kidney injury and/or dialysis requirement, myocardial infarction, cardiac arrest, cerebrovascular accident (CVA), and a composite variable of overall serious complications. Length of stay (LOS), 30-day readmission, reoperation, mortality, and withdrawal of care rates were assessed. Excess LOS was defined as LOS greater than the 75th percentile, resulting in our definition being discharge from hospital on post-operative day 14 or later. Missing data are described in Supplemental Tables S1 and S2.

Statistical analysis involved presenting categorical variables as absolute numbers and percentages, analyzed via Chi-squared tests. Continuous variables were reported as weighted means with standard deviations, using independent two-sample *t*-tests for parametric data or the Kruskal–Wallis test for non-parametric data.

To account for differences between groups and evaluate the independent effect of octogenarians on 30-day serious complications in patients undergoing esophagectomy, we developed a non-parsimonious multivariable logistic regression model using a hypothesis-driven purposeful selection approach. Additionally, a multivariate linear regression model was constructed to assess the independent effect of drains on length of stay (LOS). Bivariate analysis of variables with a *p*-value < 0.1, along with those previously identified as clinically relevant to our primary outcome, was used to create a preliminary main effects model. Model performance was assessed using the Brier score and Receiver Operating Characteristic (ROC) curve. Statistical analyses were conducted using STATA 19 software (StataCorp., College Station, TX, USA).

## 3. Results

### 3.1. Study Population

We identified a total of 11,210 cases with 10,763 cases < 80 years old and 447 cases > 80 years old (octogenarian cohort). An overwhelming majority of the patients received esophagectomy for indications related to malignancy versus 6.59% for non-malignancy or unknown indications. The median age was 65 (range 18–79) for the <80 cohort and 82 (range 80–90) for the octogenarian cohort. The sex distribution was not significantly different between the two groups with 79.94% male in the <80-year-old group and 78.97% among the octogenarians (*p* = 0.62). The majority of the cases were elective (94.22%). The majority of esophagectomies performed were the Ivor Lewis technique (n = 7463, 66.57%), followed by transhiatal (n = 1921, 17.14%) and McKeown (n = 1826, 16.29%).

Octogenarians were significantly less likely to smoke (6.49% vs. 23.90%, *p* < 0.001) and have obesity (*p* < 0.001), but were more likely to have hypertension (67.79% vs. 47.93%, *p* < 0.001), bleeding disorders (7.87% vs. 4.05%, *p* = 0.037) and heart failure (3.13% vs. 1.40%, *p* = 0.003). There was no significant difference between cohorts in rates of COPD, ascites, ventilator dependence, or diabetes. Patient demographics are summarized in Table 1.

**Figure 1:**
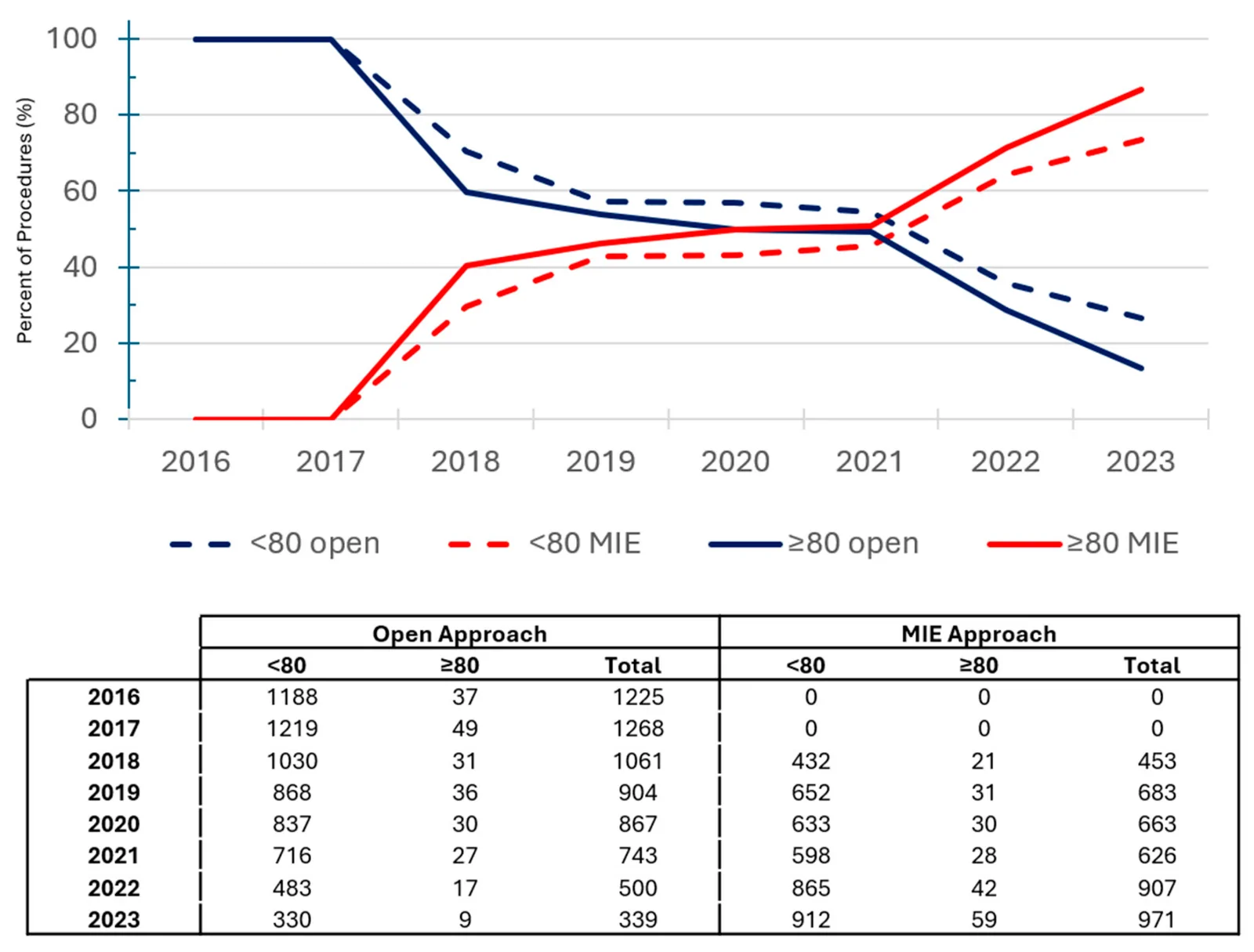
Trends in adoption of MIE techniques in esophagectomy from 2016 to 2023.

NSQIP began capturing minimally invasive esophagectomy (MIE) as of 2018, therefore all esophagectomies recorded prior to 2018 were performed via an open approach. Following its introduction in 2018, MIE accounted for 29.92% of cases in 2018 and increased rapidly to 74.12% by 2023. Among octogenarians, uptake was even greater, with 86.76% of all esophagectomies performed using minimally invasive techniques (Figure 1).

### 3.2. Univariate Analysis

**Figure 2:**
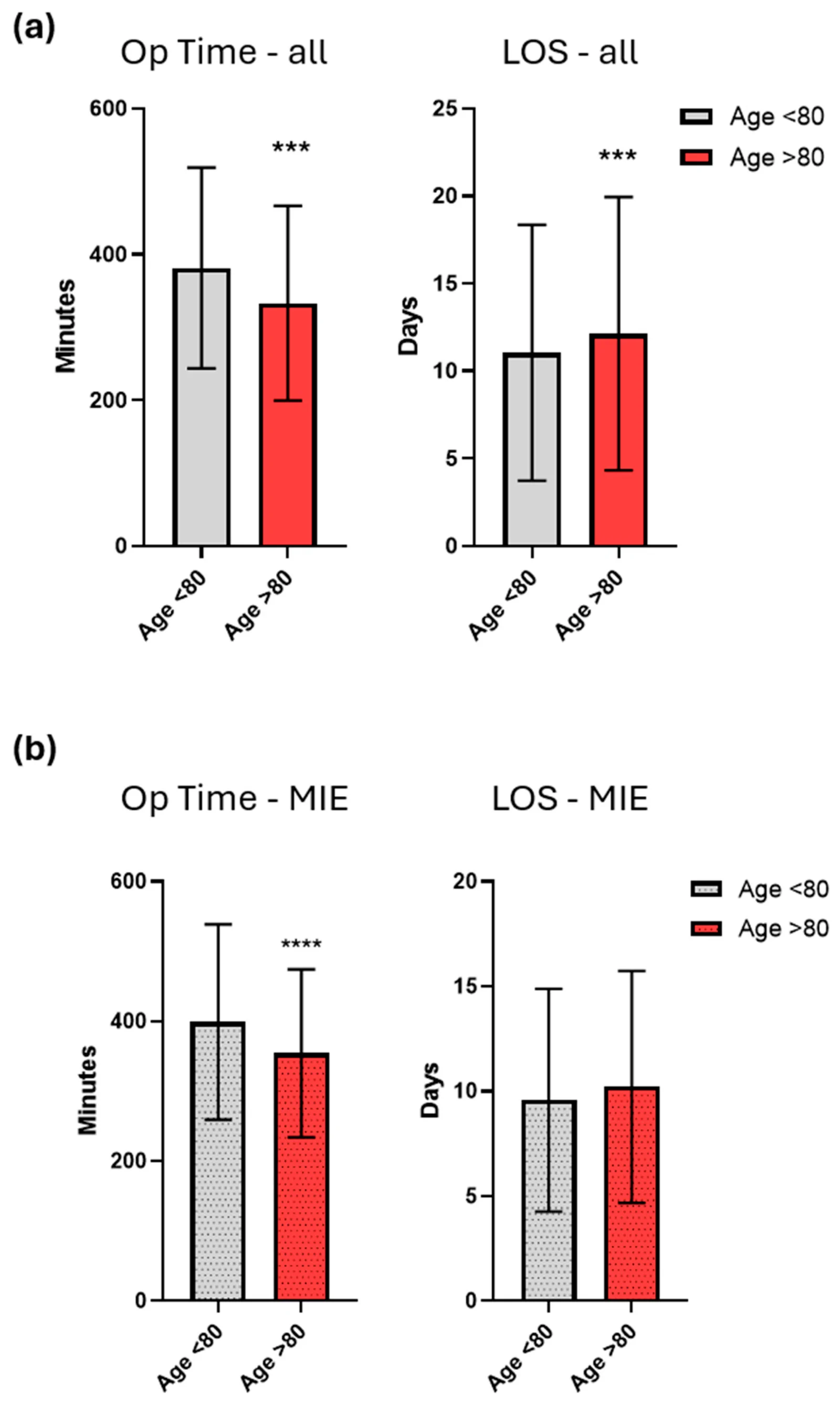
Operative time and LOS in (**a**) all esophagectomy and (**b**) MIE between patients above and below 80 years of age. (*** *p* < 0.001, **** *p* < 0.0001).

Compared to octogenarians, <80 yo patients experienced significantly longer operative time (median 370 vs. 319 min, *p* = 0.0001) while octogenarians required longer length of stay (mean 12.1 vs. 11.0, *p* = 0.0001) (Figure 2a). There were no differences in infectious complications with comparable rates of superficial, deep, and organ space surgical site infections between the two groups. The incidence of anastomotic leak was also similar between the two groups (11.47% vs. 13.20%, *p* = 0.261). Urinary tract infections were more common in the octogenarian cohort versus <80 yo cohort (3.13% versus 1.6%, *p* = 0.013), along with an increased incidence of septic shock (7.83% vs. 5.49%, *p* = 0.035). There was no difference in incidences of pneumonia between the two age groups; however, octogenarians were significantly more likely to require intubation during the postoperative period (*p* = 0.002). Octogenarians exhibited increased incidence for venous thrombosis events (VTEs, *p* < 0.01) but did not have an increased incidence of pulmonary embolisms (*p* = 0.15). There were no differences in renal insufficiency (*p* = 0.806) nor postoperative dialysis requirements (*p* = 0.411). There was no significant difference in incidence of myocardial infarctions (*p* = 0.411) or cardiopulmonary resuscitation following cardiac arrest (*p* = 0.158). Octogenarians were more likely to require blood transfusions with 13.2% versus 9.6% in the <80 yo group (*p* = 0.012). Overall, there was a significant increase in serious complications amongst octogenarians (45.86% vs. 37.95%, *p* = 0.001) (Table 2).

Rate of reoperation was not significantly different between the two age groups (*p* = 0.993); however, there was a significantly elevated rate of readmission in octogenarians (15.21% vs. 11.06%, *p* = 0.006). There was also an overall 1.8-fold increase in mortality with 5.37% in octogenarians vs. 2.97% (*p* = 0.004) in those below 80.

Subset analysis limited to MIE (laparoscopic or robotic approaches) showed similar trends in octogenarians with significant increase in rates of readmission (18.95% vs. 11.63%, *p* = 0.001), serious complications (47.39% vs. 35.70%, *p* = 0.001) and discharge to higher level of care (17.54% vs. 9.26%, *p* < 0.001) (Table 3). There is a trend towards increased mortality in octogenarians as well (4.27% vs. 2.2%, *p* = 0.051). There remains no significant difference in anastomotic leak (16.11% vs. 12.78%, *p* = 0.16) or reoperation (15.17% vs. 16.03%, *p* = 0.738). Unique to MIE, octogenarians did not exhibit significantly increased LOS (mean of 10.21, SD 5.53 vs. mean of 9.57 SD 5.31, *p* = 0.116). Operative time remains significantly shorter for octogenarians (mean of 354.2 ± 130.1 min vs. mean of 399.0 ± 139.7 min, *p* = 1.304 × 10^−6^) (Figure 2b).

### 3.3. Multivariate Analysis

Multivariate analysis was performed on outcomes of interest including mortality, complications, extended LOS, reoperation, readmission, and discharge to higher care, as well as anastomotic leak (Table 4). With multivariable logistic regression models, an age over 80 was found to be independently associated with discharge to higher care (OR 2.56, CI 1.83–3.60, *p* < 0.001), readmission (OR 1.48, CI 1.08–2.03, *p* = 0.015), and serious complications (OR 1.36, CI 1.082–1.715, *p* = 0.008), as well as extended LOS (OR 1.54, CI 1.20–1.98 *p* = 0.001). However, it was not significantly associated with reoperation (OR 0.94, CI 0.68–1.28, *p* = 0.686) and did not pass the significance threshold for 30-day mortality (OR 1.74, CI 0.97–3.13, *p* = 0.065). 

Other patient characteristics that were significantly associated with 30-day mortality included ASA 3+ (OR 2.98 CI 1.57–5.68, *p* = 0.001), CHF (OR 2.37 CI 1.07–5.28, *p* = 0.034), COPD (OR 1.88 CI 1.26–2.82, *p* = 0.002), smoking history (OR 1.42 CI 1.04–1.94, *p* = 0.029), and diabetes (OR 1.38 CI 1.12–1.70, *p* = 0.003). The aforementioned mortality risk factors were also associated with extended LOS but only diabetes was associated with increased odds of readmission (OR 1.36 CI 1.22–1.53, *p* < 0.001) along with being octogenarians (OR 1.48 CI 1.08–2.03, *p* = 0.015). Many factors were significantly associated with increased odds of anastomotic leak including BMI (OR 1.07, CI 1.02–1.13, *p* = 0.01), ASA 3+ (OR 1.26 CI 1.04–1.52, *p* = 0.02), diabetes (OR 1.21 CI 1.09–1.34, *p* < 0.001), and smoking history (OR 1.28 CI 1.11–1.48, *p* = 0.001).

Interestingly, BMI was protective against extended LOS (OR 0.92 CI 0.87–0.96, *p* < 0.001), but did not affect readmission, reoperation, serious complications, or mortality, while female sex was associated with decreased odds of anastomotic leak (OR 0.83, CI 0.71–0.98, *p* = 0.024) but also with extended LOS (OR 1.14 CI 1.00–1.30, *p* = 0.045).

Prior treatment with radiation therapy with 90 days of surgery did not appear to significantly impact patients’ surgical outcome in anastomotic leak, readmission, reoperation or 30-day mortality but did increase the patients’ odds of discharge to a higher level of care (OR 1.35 CI 1.03–1.77, *p* = 0.031) and decrease odds of extended LOS (OR 0.82 CI 0.69–0.97 *p* = 0.024). Meanwhile, chemotherapy was protective against extended LOS (OR 0.70 CI 0.59–0.84, *p* < 0.001), discharge to higher level of care (OR 0.62 CI 0.47–0.82, *p* = 0.001) and reoperation (OR 0.77 CI 0.63–0.94 *p* = 0.012) without significant effect on rate of 30-day mortality (*p* = 0.451).

## 4. Discussion

Esophageal cancer is increasingly a disease of older adults, with incidence peaking in the eighth decade of life [3]. As the population ages, octogenarians have become a clinically important group in whom surgical decision-making is often complicated by advanced age, comorbidity burden, and uncertainty regarding operative risk and expected outcomes, making patient counseling especially challenging. Herein, we present the largest contemporary analysis using the ACS-NSQIP database outlining outcomes of open and minimally invasive esophagectomy in this population, filling this critical gap in the literature. Prior studies in smaller cohorts or single-center studies have yielded conflicting results [4,12,13]. Over the past two decades, esophagectomy has evolved substantially, with the Ivor Lewis technique becoming the dominant approach as well as the adoption of minimally invasive techniques [15].

Using the ACS-NSQIP database from 2016 to 2023, we demonstrate that octogenarians have an overall mortality of nearly double that of the <80 yo cohort (5.37% vs. 2.97%). This was in line with previous multi-center studies of 4–5% [7,18,19]. The effect was not statistically significant (*p* = 0.065) in multivariate analysis with odds ratio of 1.73. This suggests that other variables such as patient factors and comorbidities may be stronger predictors of post-operative mortality than age. Indeed, on multivariate analysis, elevated ASA (3+), diabetes, history of smoking, COPD, and CHF all posed significantly increased odds of mortality. Interestingly, while the octogenarians were generally expected to be more comorbid, this was not always reflected in the esophagectomy patients. While octogenarians did exhibit higher rates of comorbidities including hypertension, bleeding disorders and heart failure, they were significantly less likely to smoke and less obese. There was also no significant difference in other comorbidities such as COPD, diabetes, or ascites. It has also long been described that frailty is a more accurate predictor than age in post-operative outcomes [20,21,22]. Overall, 98.81% of all esophagectomy patients were functionally independent prior to surgery. Of the octogenarians, only 6 patients (1.34%) were partially dependent, and none were fully dependent. The <80 yo cohort exhibited a similar trend with only 0.93% of patients with partially functional dependency and 0.12% were totally dependent. Given the observational nature of the study, it is likely that surgeons exercised selection bias in choosing eligible patients with stricter criterion applied in the aged population.

Despite advancements in surgical technique and the adoption of minimally invasive procedures, esophagectomy remains associated with high rates of postoperative morbidity. The present study revealed an overall rate of serious complication at 38.27% with octogenarians at significantly higher risk at 45.86%. Previous studies that examined data from Esophagectomy Complications Consensus Group (ECCG) also described postoperative morbidity of 59–65% [7,23]. Pulmonary complication is one of the most common adverse events following esophagectomy [24]. Intriguingly, NSQIP data did not show a significant change in rate of postoperative pneumonia in octogenarians; however, there was significant increase in unplanned intubations (*p* = 0.0019). This is in contrast with previous studies which showed a higher rate of post-esophagectomy pneumonia in octogenarians [14]. The increased morbidity is also reflected in LOS post-operatively with significantly increased mean length of stay by 1 day (12.13 vs. 11.04 days, *p* = 0.0001).

Despite the increase in morbidity and mortality, surgical parameters for octogenarians did not reflect the same adverse trends. In fact, octogenarians were found to have significantly shorter mean operative time by nearly 48 min (*p* = 0.0001). Possibly due to decreased rate of obesity which has been shown to correlate with operative time [25]. Previous literature has shown that operative time is a significant predictor of poor post-operative morbidity [26]. This could be an indication that the outcomes of the octogenarian group are secondary to the patients’ comorbidities rather than the operative challenges of an elderly population.

Of the surgical approaches outlined, McKeown was significantly correlated with increased rate of complications on multivariate analysis with increased rate of anastomotic leak, reoperation and serious complications whereas transhiatal approach did not show the same trends (Table 5). Another study on NSQIP data has previously identified that McKeown approach is the least common approach used at under 20% of esophagectomies and continues to decline over time [15]. This is reflective of the epidemiological shift from squamous cell carcinoma of the esophagus which is predominantly in proximal to middle third of the esophagus to adenocarcinoma of the esophagus commonly in distal esophagus and gastroesophageal junction [27]. Ivor Lewis is the more appropriate technique for cancers of the distal esophagus/gastroesophageal junction and expectedly rose in prominence. The same epidemiological shift was not observed in Asian and African countries where squamous cell carcinoma of esophagus remains the overwhelming majority (~90%) of all esophageal cancers and McKeown remains the dominant surgical approach [2]. The epidemiological divide between Western and Asia/African nations limits the cross-applicability of our findings.

Minimally invasive techniques also appear to have a positive impact on patient outcomes with both laparoscopic and robotic approaches significantly decreasing the length of stay and odds of complications even when accounting for comorbidities on multivariate analysis.

We observed a rapidly shifting trend toward MIE since being tracked by the NSQIP database, with 29.92% of cases (453 MIE procedures) being performed minimally invasive (laparoscopic or robotic) in 2018, compared to 74.12% of total cases (971 MIE procedures) by 2023. Unfortunately, NSQIP does not differentiate between hybrid vs. full MIE/robotic approaches, thus hybrid procedures were analyzed as MIE/robotics where applicable. There is a slightly higher adoption rate of MIE in octogenarians (86.76%) vs. <80 yo cohort (73.43%) as of 2023. Within the MIE cohort, octogenarians no longer exhibit significant difference in LOS (*p* = 0.116) and 30-day mortality rate is borderline non-significant (*p* = 0.051). There remains a significant increase in readmission, serious complications, and discharge to higher level of care within the octogenarian population. Previous studies have demonstrated that adoption of MIE techniques significantly decreased morbidity and mortality of patients and indeed our results show early promise of MIE approaches in tightening the outcome gap between octogenarians and <80 yo patients. This finding may have a significant impact on treatment decisions for elderly patients. Previous study comparing esophagectomy versus chemoradiotherapy showed survival advantage in patients age 75–79 but not in octogenarians [28]. However, that study was carried out using data from 2008 to 2011 prior to the adoption of minimally invasive techniques. The improvement in octogenarians undergoing MIE may provide a benefit over chemoradiotherapy alone and future updates may be needed to delineate the outcome between the two in the elderly population. While the findings should be interpreted in the lens of the highly selected patient population, further studies exploring outcomes of surgical techniques in matched populations could be another avenue by which esophagectomies may be optimized.

## 5. Conclusions

We present the first and largest study to evaluate esophagectomy outcomes in octogenarians since the widespread adoption of MIE. Overall, this study concludes that the current octogenarian patient population selected for esophagectomy by surgeons, despite comparable or more favorable pre-operative comorbidities, still exhibit increased post-operative morbidity and mortality within a 30-day period. However, the adoption of minimally invasive techniques is beginning to narrow the gap in outcomes and future studies may be warranted to reassess the risk of esophagectomy in octogenarians compared to other treatment modalities. We identified key patient factors influencing esophagectomy outcomes that can aid in patient selection and shared decision-making in this vulnerable population.

## 6. Limitations

This study has several important limitations by nature of its design. First, the retrospective nature of the analysis predisposes data to potential selection bias by surgeons. While NSQIP only examines patients who underwent surgery, one study examining other datasets such as National Cancer Database described that only 11.5% of octogenarians with esophageal cancer underwent esophagectomy versus 33.3% of patients under the age of 80, with the same trend persisting after multi-variate adjustments [29]. This is also indirectly reflected in our study: whereas ~29% of esophageal cancer arises in patients above 75 years of age [30], only 4% of the NSQIP cohort who underwent esophagectomy were over the age of 80. This is an indication that there is intense clinical selection in octogenarians who undergo esophagectomy; the findings from this cohort may serve as a proof of feasibility and we caution against indiscriminate extrapolations to octogenarians at large. We have included esophagectomy for all indications rather than malignancy alone, though malignancy accounted for 93.41% of all included cases. Certainly, the diversity in both surgical indications and types of malignancy could introduce heterogeneity in data but the findings are reflective of the overall safety of the esophagectomy procedure in a Western setting.

Furthermore, we are only able to abstract data using the ACS-NSQIP’s pre-determined set of variables, which may not capture nuanced technical details such as anastomotic technique, extent of nodal dissection, intraoperative blood loss, robotic platform, and and others, which may confound associations between operative approach and outcomes. The database also only captures thirty-day outcomes precluding any assessment of long-term endpoints such as cancer-related survival or late deterioration or functional decline. Previous studies have identified quality-of-life measures such as functional decline and autonomy are frequently prioritized by patients over marginal survival benefits, which is not captured by NSQIP and represents an important blind spot in our data [31,32]. Finally, this cohort of patients is that of primarily functionally independent treated at participating institutions, most of which are higher-volume academic or large community hospitals, limiting generalizability to smaller or non-NSQIP centers as well as more frail or dependent populations and it is unclear if the same trends observed in this study will apply and extrapolations of data should be done cautiously.

## Figures and Tables

**Table 1:** Patient demographics and clinical characteristics.

	Age < 80		Age ≥ 80		
Age (Median: Range)	65: 18–79		82: 80–90		
	Patients	%	Patients	%	*p*-Value
Sex (male)	8604	79.94	353	78.97	0.616
Race					
Black	404	3.76	8	1.79	
White	7669	71.3	296	66.22	
Asian	322	2.99	5	1.12	
Hispanic	116	1.08	0	0	
Diabetes					0.891
insulin dependent	648	6.02	27	6.04	
Non-insulin	1433	13.31	63	14.89	
non-diabetic	8682	80.67	357	79.87	
Smoking history (within 1 year)					<0.001
Yes	2572	23.9	29	6.49	
No	8191	76.1	418	93.51	
ASA					<0.001
1	23	0.21	0	0	
2	1475	13.71	38	0.52	
3	8273	76.89	341	76.46	
4	973	9.04	66	14.8	
Functional status					0.594
independent	10,636	98.82	441	98.66	
partially	100	0.93	6	1.34	
totally dependent	13	0.12	0	0	
Obesity					<0.001
Normal	3513	32.64	166	37.14	
Overweight	3763	34.96	203	45.41	
Underweight	379	3.52	8	1.79	
Class I obesity	1983	18.42	55	12.3	
Class II obesity	737	6.85	10	2.24	
Class III obesity	388	3.6	5	1.12	
Ventilator dependent					0.145
Yes	17	0.16	2	0.45	
No	10,746	99.84	445	99.55	
COPD					0.7
Yes	774	7.19	30	6.71	
No	9989	92.81	417	93.29	
Ascites					0.275
Yes	8	0.07	1	0.22	
No	10,755	99.93	446	99.78	
Heart failure (within 30 days)					0.003
Yes	151	1.4	14	3.13	
No	10,612	98.6	433	96.87	
Hypertension					<0.001
Yes	5159	47.93	303	67.79	
No	5604	52.07	144	32.21	
Disseminated cancer					0.362
Yes	483	4.49	16	3.58	
No	10,280	95.51	431	96.42	
Immunosuppression					0.514
Yes	416	3.87	20	4.47	
No	10,347	96.13	427	95.53	
Bleeding disorder					0.037
Yes	105	4.05	10	7.87	
No	2485	95.95	117	92.13	
Fall within 6 months					<0.001
Yes	20	0.51	12	6.59	
No	440	11.27	156	85.71	

**Table 2:** Univariate analysis of clinical outcomes in all esophagectomies between young (<80) and elderly (≥80) patients.

		Age < 80	Age ≥ 80	
	Total (n)	n (%)	n (%)	*p*-Value
Wound complications	1865	1798 (16.71%)	67 (14.99%)	0.34
UTI	186	172 (1.6%)	14 (3.13%)	0.013
Pneumonia	1712	1633 (15.18%)	79 (17.67%)	0.1498
Septic shock	626	591 (5.49%)	35 (7.83%)	0.0348
C diff infection	175	168 (1.56%)	7 (1.57%)	0.993
Blood transfusion	1092	1033 (9.6%)	59 (13.2%)	0.012
VTE requiring therapy	276	255 (2.37%)	21 (4.7%)	0.0019
Stroke/CVA	30	30 (0.28%)	0 (0%)	0.264
PE	222	209 (1.94%)	13 (2.91%)	0.1507
Intubation	1163	1097 (10.19%)	66 (14.77%)	0.0019
Renal insufficiency	166	160 (1.69%)	6 (1.53%)	0.806
Post-op dialysis	119	116 (1.08%)	3 (0.67%)	0.411
MI	135	128 (1.19%)	7 (1.57%)	0.474
MI requiring CPR	183	172 (1.6%)	11 (2.46%)	0.1584
reoperation	1804	1732 (16.09%)	72 (16.11%)	0.993
readmission	1258	1190 (11.06%)	68 (15.21%)	0.006
Anastomotic leak	1293	1234 (11.47%)	59 (13.20%)	0.261
Death	344	320 (2.97%)	24 (5.37%)	0.004
Withdrawal of Care	117	106 (1.97%)	11 (4.55%)	0.006
Serious complications	4290	4085 (37.95%)	205 (45.86%)	0.001

**Table 3:** Univariate analysis of clinical outcomes in MIE between patients above and below 80 years of age.

		Age < 80	Age ≥ 80	
	Total	n (%)	n (%)	*p*-Value
30 d mortality	99	90 (2.2%)	9 (4.27%)	0.051
Readmission	516	476 (11.63%)	40 (18.95%)	0.001
Reoperation	688	656 (16.03%)	32 (15.17%)	0.738
Anastomotic leak	557	523 (12.78%)	34 (16.11%)	0.16
Serious complications	1561	1461 (35.70%)	100 (47.39%)	0.001
Excessive LOS	880	825 (20.16%)	55 (26.07%)	0.038
Higher Level of Care	416	379 (9.26%)	37 (17.54%)	<0.001

**Table 4:** Multivariate analysis of patient factors associated with clinical outcomes in all esophagectomies.

	30 Day Mortality		Reoperation		Readmission		Serious Complication		Extended LOS		Higher Level of Care		Anastomotic Leak	
	Odds Ratio	*p*-Value	Odds Ratio	*p*-Value	Odds Ratio	*p*-Value	Odds Ratio	*p*-Value	Odds Ratio	*p*-Value	Odds Ratio	*p*-Value	Odds Ratio	*p*-Value
Octogenarian	1.737 (0.966–3.126)	0.065	0.937 (0.681–1.287)	0.686	1.481 (1.081–2.03)	0.015	1.362 (1.082–1.715)	0.008	1.54 (1.197–1.982)	0.001	2.568 (1.832–3.6)	<0.001	1.232 (0.912–1.664)	0.173
Sex (female)	1.24 (0.886–1.736)	0.210	0.98 (0.842–1.141)	0.798	1.093 (0.919–1.299)	0.313	1.044 (0.929–1.173)	0.468	1.143 (1.003–1.303)	0.045	1.059 (0.872–1.286)	0.560	0.832 (0.709–0.976)	0.024
BMI	1.012 (0.899–1.138)	0.846	1.002 (0.951–1.056)	0.937	1.03 (0.97–1.093)	0.341	1.015 (0.974–1.057)	0.484	0.916 (0.873–0.96)	<0.001	0.952 (0.891–1.018)	0.147	1.072 (1.017–1.129)	0.01
ASA 3+	2.984 (1.568–5.681)	0.001	1.211 (1.005–1.458)	0.044	1.037 (0.842–1.276)	0.734	1.402 (1.219–1.612)	<0.001	1.51 (1.277–1.786)	<0.001	2.045 (1.536–2.723)	<0.001	1.255 (1.036–1.520)	0.02
Diabetes	1.375 (1.115–1.695)	0.003	1.168 (1.053–1.295)	0.003	1.364 (1.221–1.525)	<0.001	1.244 (1.145–1.351)	<0.001	1.224 (1.116–1.343)	<0.001	1.243 (1.094–1.413)	0.001	1.210 (1.091–1.340)	<0.001
Smoking history	1.418 (1.036–1.941)	0.029	1.331 (1.156–1.533)	<0.001	1.024 (0.863–1.214)	0.787	1.32 (1.18–1.476)	<0.001	1.278 (1.127–1.45)	<0.001	1.053 (0.874–1.269)	0.584	1.28 (1.107–1.48)	0.001
COPD	1.883 (1.259–2.817)	0.002	1.206 (0.97–1.499)	0.092	1.026 (0.784–1.342)	0.853	1.644 (1.377–1.963)	<0.001	1.752 (1.452–2.113)	<0.001	1.679 (1.301–2.168)	<0.001	1.104 (0.879–1.386)	0.395
CHF	2.374 (1.068–5.277)	0.034	1.549 (0.979–2.452)	0.061	0.973 (0.538–1.763)	0.929	1.695 (1.133–2.536)	0.010	1.83 (1.198–2.795)	0.005	1.338 (0.749–2.388)	0.325	1.203 (0.741–1.953)	0.456
Chemotherapy within 90d	0.826 (0.503–1.357)	0.451	0.77 (0.628–0.944)	0.012	1.071 (0.846–1.357)	0.567	0.808 (0.692–0.945)	0.007	0.7 (0.587–0.835)	<0.001	0.622 (0.471–0.821)	0.001	0.966 (0.787–1.184)	0.736
Radiotherapy within 90d	1.278 (0.793–2.059)	0.314	0.868 (0.713–1.057)	0.159	0.966 (0.773–1.206)	0.758	0.907 (0.782–1.051)	0.194	0.822 (0.693–0.974)	0.024	1.349 (1.028–1.77)	0.031	0.862 (0.71–1.047)	0.135
Cancer	0.985 (0.688–1.411)	0.935	0.893 (0.766–1.041)	0.147	1.027 (0.854–1.235)	0.779	0.953 (0.844–1.076)	0.434	0.856 (0.748–0.98)	0.024	1.031 (0.844–1.26)	0.762	0.936 (0.798–1.098)	0.417

**Table 5:** Multivariate analysis of surgical parameters associated with clinical outcomes in all esophagectomies.

	30 Day Mortality		Reoperation		Readmission		Serious Complication		Extended LOS		Higher Level of Care	
	Odds Ratio	*p*-Value	Odds Ratio	*p*-Value	Odds Ratio	*p*-Value	Odds Ratio	*p*-Value	Odds Ratio	*p*-Value	Odds Ratio	*p*-Value
Op Time	1.001 (1–1.002)	0.044	1.001 (1.001–1.002)	<0.001	1.001 (1–1.001)	0.012	1.001 (1.001–1.002)	<0.001	1.002 (1.002–1.003)	<0.001	1.001 (1.001–1.002)	<0.001
Laparoscopic	0.703 (0.484–1.022)	0.065	1.038 (0.889–1.213)	0.636	1.172 (0.983–1.396)	0.076	0.893 (0.793–1.005)	0.060	0.564 (0.491–0.648)	<0.001	0.881 (0.715–1.085)	0.233
Robotic	0.749 (0.407–1.378)	0.353	0.963 (0.746–1.244)	0.775	0.922 (0.685–1.24)	0.592	0.756 (0.621–0.921)	0.005	0.425 (0.335–0.54)	<0.001	0.763 (0.517–1.125)	0.171
Transhiatal	1.242 (0.85–1.816)	0.263	1.14 (0.955–1.36)	0.146	0.991 (0.805–1.218)	0.928	1.11 (0.97–1.27)	0.131	1.047 (0.896–1.223)	0.566	1.106 (0.881–1.39)	0.384
McKeown	0.972 (0.664–1.423)	0.883	1.384 (1.178–1.626)	<0.001	1.083 (0.891–1.315)	0.424	1.308 (1.149–1.489)	<0.001	1.028 (0.89–1.187)	0.709	1.19 (0.969–1.462)	0.097
Year	0.964 (0.893–1.04)	0.345	0.987 (0.954–1.021)	0.446	1.002 (0.964–1.042)	0.911	1.014 (0.989–1.04)	0.280	1.028 (0.998–1.058)	0.064	0.83 (0.794–0.867)	<0.001

## Data Availability

The data presented in this study are openly available in [NSQIP] [https://www.facs.org/quality-programs/data-and-registries/acs-nsqip/] (accessed on 30 April 2025).

## Supplementary Materials

The following supporting information can be downloaded at: https://www.mdpi.com/article/10.3390/jcm15197590/s1, Supplemental Table S1: Number of patients with reported data for each of the patient characteristic variables included in Table 1. Supplemental Table S2: Number of patients with reported data for each of the variables in univariate analysis included in Table 2.
