# Supplementary material for: Esophagectomy in Octogenarians in the Minimally Invasive Era: A NSQIP Analysis of Surgical Outcomes
Source: J Clin Med. 2026 Sep 30;15(19):7590. doi: 10.3390/jcm15197590 (PMC13666228; doi:10.3390/jcm15197590)
Supplement: Supplementary file 1 [file jcm-15-07590-s001.zip › jcm-4511478-supplementary.pdf]

Supplemental Table S1. Number of patients with reported data for each of the patient characteristic variables included in Table 1.

Age (median: range)	Age <80<br>65: 18-79		Age≥80<br>82: 80-90	
	Patients	% reported	Patients	% reported
**Sex**	8511	79.1	309	69.1
**Race**	10763	100	447	100
**Diabetes**	10763	100	447	100
**Smoking history**	10763	100	447	100
**ASA**	10744	99.8	447	100
**Functional status**	10749	99.9	447	100
**Obesity**	10763	100	447	100
**Veiltator dependent**	10763	100	447	100
**COPD**	10763	100	447	100
**Ascites**	10763	100	447	100
**Heart failure**	10763	100	447	100
**Hypertension**	10763	100	447	100
**Disseminated cancer**	10763	100	447	100
**Bleeding disorder**	2590	24.1	127	28.4
**Fall within 6 months**	460	4.27	168	37.6

Supplemental Table S2. Number of patients with reported data for each of the variables in univariate analysis included in Table 2.

Age (median: range)	Age <80<br>65: 18-79		Age≥80<br>82: 80-90	
	Patients	% reported	Patients	% reported
Wound complications	10763	100	447	100
UTI	10763	100	447	100
Pneumonia	10763	100	447	100
Septic shock	10763	100	447	100
C diff infection	10763	100	447	100
Blood transfusion	10763	100	447	100
VTE requiring therapy	10763	100	447	100
Stroke/CVA	10763	100	447	100
PE	10763	100	447	100
Intubation	10763	100	447	100
Renal insufficiency	9449	87.8	392	87.7
Post-op dialysis	10763	100	447	100
MI	10763	100	447	100
MI requiring CPR	10763	100	447	100
reoperation	10763	100	447	100

readmission	10763	100	447	100
Anastomotic leak	10763	100	447	100
Death	10763	100	447	100
Withdrawal of Care	5374	49.9	242	54.1
